# Crossing silos: how changes in EU chemicals policy and legislation are reflected in its pharmaceutical policy and legislation

**DOI:** 10.1080/20523211.2025.2587439

**Published:** 2025-11-24

**Authors:** Mirella Miettinen

**Affiliations:** UEF Law School, Joensuu, Finland

**Keywords:** Chemicals, circular economy, environment, green transformation, transformation governance, pharmaceuticals, value chains

## Abstract

**Background:**

The European Union (EU) has introduced several changes to its chemicals policy and legislation with an ambition to transform society greener. This study examined selected changes introduced in EU chemicals policy and legislation and how they may affect the pharmaceutical sector. The objective was to find out whether structural changes in one political sphere (chemicals) influence the other political sphere (health).

**Methods:**

First, concrete changes to EU chemicals legislation or its implementation were identified. Then, qualitative content analysis was used to analyse how these changes are reflected in EU pharmaceutical policy, legislation and related guidance documents. Data was analysed using both deductive and inductive approaches. The concrete changes identified were used as codes for the deductive classification of quotations. The analysis continued inductively by examining these quotations for the topics they raise in EU pharmaceutical policy, legislation and related guidance.

**Results:**

The results imply that structural changes in EU political sphere of chemicals may have a range of implications for the pharmaceutical sector. The need for derogations in certain cases regarding medicinal products was recognised, but in general, the pharmaceutical sector will not be exempted from the application of the new provisions. However, changes to EU chemicals legislation were rarely referred to in pharmaceutical policy, legislation and guidance. An inductive analysis of the quotations revealed that some changes (such as substances with endocrine-disrupting properties and changes to EU water legislation) have got more prominence in the pharmaceutical sector than others.

**Conclusion:**

The analysis indicated that the pharmaceutical sector is taking a defensive approach to changes in EU chemicals legislation. This may be partly because many changes occur simultaneously, creating uncertainty about their combined and cumulative impacts. Closer cooperation between the Environment and Health Directorates-General is needed to steer more coherent transformation governance across different policy sectors in the EU.

## Background

Under von der Leyen’s first Commission (2019–2024), the European Union (EU) embarked on an ambitious path to make society greener. The European Green Deal (EGD) included a roadmap of the policies and measures to address this goal (European Commission [EC], 2019a). The EC stated that these measures also target pollution from ‘new or particularly harmful sources of pollution such as micro plastics and chemicals, including pharmaceuticals’ (EC, 2019a, p. 14), and stressed that the protection and restoration of natural ecosystems, the sustainable use of natural resources, and the improvement of human health should be a priority in the transformational change. Many measures announced in the EGD or in accompanying policies, which aim to promote green transformation, have been taken forward. The objectives include pursuing a toxic-free environment, improving cooperation between EU agencies, and accelerating the clean and circular economy (CE).

The concept of ‘green transformation’ is not firmly consolidated. There is interpretative flexibility and little consensus on its conceptual basis (Amundsen & Hermansen, 2021; Feola, 2015). However, the general idea of transformation as a fundamental shift – opposed to incremental changes – towards sustainability to address global environmental change caused by human activities is widely accepted (Feola, 2015). Related concepts, such as ‘transition’ also overlap with transformation. Stirling (2015) explains that transitions are typically managed within controlled structures towards a particular known outcome, whereas transformations involve more diverse political alignments that challenge dominant structures and entail tacit social knowledge in pursuit of competing, even unknown, ends. It is likely that multiple transformations take place simultaneously, overlapping and intersecting in unpredictable ways (Scoones et al., 2015). Soininen et al. (2025) discuss the differences between transition governance and transformation governance and note that examples of transition governance include governmental collaboration with companies and civil society to promote the development and uptake of new technological or social innovations, while transformation governance focuses on deliberate and non-linear societal change. O’Brien (2018) states that it is important to consider how deliberate societal transformations occur and that three interacting spheres – the practical, political and personal – should be taken into account to be able to capture the breadth and depth of the needed transformation. The personal sphere represents ‘the subjective beliefs, values, worldviews and paradigms’, the practical sphere includes ‘actions, interventions, strategies and behaviors that directly contribute to a desired outcome’, and the political sphere contains ‘the systems and structures that facilitate or constrain practical responses’ (O’Brien, 2018, pp. 155, 156). O’Brien explains that structures are the norms, rules, regulations, institutions, regimes and incentives that influence how larger systems are designed, organised and governed. The introduction of regulation and other structural incentives at regional level (e.g. the EU) is one way to implement transformation governance (Soininen et al., 2025).

In the field of health, the COVID-19 pandemic and the ever-growing threat to global health posed by antimicrobial resistance (AMR) have prompted different governance sectors to seek more integrated approaches to solving these problems. This requires closer cooperation between the health sector (both human and animal) and the environmental sector. Traditionally, environmental considerations have been largely absent from the literature on EU pharmaceutical legislation. In 2015, Hervey and McHale were not aware of any source linking EU environmental legislation to EU health law. As pharmaceuticals are regulated by specific legislation, they are also rarely discussed in the literature on EU environmental law (Van Carlster & Reins, 2017). However, this siloed approach is changing. For example, the specific objectives of the latest EU Health Programme (Regulation (EU) 2021/522) include supporting sustainable and environmentally friendly production and supply chains and developing pharmaceuticals that are less harmful to the environment. Bazzan (2020) asserts that the EU Health Programme, like the EGD, has been adopted to address the inadequacy of fragmented policy responses to cross-cutting issues (such as the COVID-19 pandemic). Bazzan analysed the manifestation of the four dimensions of policy integration – policy frame, subsystem involvement, policy goals, policy instruments – in the EU Health Programme and found that integration was only moderate in terms of subsystem involvement and consistency of policy mixes. Subsystems refer to, for example, food safety, trade policies, and environmental policies. She argues that at EU level this is partly due to the siloed approach between different Directorates-General and their respective agencies.

Although there are an increasing number of publications dealing with sustainability aspects in the pharmaceutical sector (De Spiegeleer & Wynendaele, 2025), for example the implications of the EGD for EU pharmaceutical policy and legislation are underexplored. De Spiegeleer and Wynendaele (2025) emphasise that the lifecycle of a medicine is significantly shaped by legal frameworks and note that scientific publications on sustainability topics (also in the pharmaceutical sector) increased sharply after the 2019 EGD. They also provide an overview of EU policies (besides the EGD) that interact with the pharmaceutical sector. As the EC has introduced several changes to its chemicals policy and legislation in pursuit of green transformation, it is timely to analyse their linkages with pharmaceutical policy and legislation, particularly regarding the EGD.

This study analyses selected changes introduced in EU chemicals policy and legislation (mainly through the EGD) and how they are reflected in EU pharmaceutical policy and legislation. The specific research questions are: (1) Which changes in EU chemicals policy and legislation that support green transformation may affect the pharmaceutical sector; and (2) How are these changes reflected in EU pharmaceutical policy, legislation and related guidance? The objectives of the research are to find out whether these structural incentives introduced in the political sphere (O’Brien, 2018) in one sector (chemicals) of the EU policy influence the political sphere of the other sector (health), and to contribute to the discussion on transformation governance towards a greener society in these sectors.

## Methods

### Analysis of EU chemicals policy documents

The analysis began with a targeted (selective) manual search of EU chemicals policy documents. The documents were first selected from those included in the Annex of the EGD (EC, 2019b). Not all policy documents listed in the EGD Annex were included; for example, climate, energy and transport policies were omitted to narrow the scope of the study. The aim was not to conduct a comprehensive search of all policy documents, but to select a reasonable set of them for analysis. Documents were included if they were considered to contain initiatives aimed at promoting green transformation (e.g. striving for a toxic-free environment or fostering the CE) and which could affect the pharmaceutical sector. In addition, some policy documents were included that were named in the documents listed in the EGD Annex. The Communication on an EU framework on endocrine disruptors (EC, 2018) was also included, although it was adopted before the EGD. Consequently, 20 documents were examined for the relevant initiatives (see Supplemental Table S1). The initiatives were further examined to identify concrete changes made or proposed to EU chemicals legislation or its implementation that may have an impact on the pharmaceutical sector.

### Analysis of EU pharmaceutical policy documents, legislation and related guidance

Qualitative content analysis (Krippendorff, 2019) was used to analyse how the concrete changes to EU chemicals legislation or its implementation are reflected in EU pharmaceutical policy and legislation. ATLAS.ti software was used (Atlas.ti Scientific Software Development GmbH, 2024). Eight pharmaceutical policy documents and twelve pharmaceutical legislation and guidance documents were examined (see Supplemental Table S2). The documents were selected manually based on their relevance to the changes listed in Table 1. The concrete changes identified were used as codes in the Atlas.ti for the deductive classification of quotations. The analysis was continued inductively by examining these quotations for the topics they address to answer research question two.

**Table 1.** Concrete changes made or proposed to EU chemicals legislation or its implementation.

**Group 1: Amendments to REACH**	
G1.1	Commission Regulation (EU) 2021/2045 amended Annex XIV of REACH by adding bis(2-ethylhexyl) phthalate (DEHP), benzyl butyl phthalate (BBP), dibutyl phthalate (DBP) and diisobutyl phthalate (DIBP) due to their endocrine-disrupting effects on human health (and on the environment for DEHP) the exemptions from the authorisation requirements for immediate packaging of medicinal products were removed from DEHP, BBP and DBP
G1.2	Commission Regulation (EU) 2023/2055 amended Annex XVII of REACH to include synthetic polymer microparticles under REACH restrictions human and veterinary medicinal products are exempted; reporting requirements apply from 2027 restrictions apply to medical devices from 2029
G1.3	The Restrictions Roadmap^a^ prioritises endocrine disruptors; carcinogenic, mutagenic and reprotoxic substances (CMRs); persistent, bioaccumulative and toxic (PBT) and very persistent and very bioaccumulative (vPvB) substances; immunotoxicants, neurotoxicants, substances toxic to specific organs and respiratory sensitisers for (group) restrictions under REACH
G1.4	Proposal for a restriction of PFASs^b^ covers all uses, but derogations are proposed for PFASs used as active substances (but not as co-formulants) in human and veterinary medicinal products derogation includes a reporting requirement for manufacturers and importers of active substances
**Group 2: EU water and industrial emissions legislation**	
G2.1	Commission implementing decision (EU) 2022/1307^c^ added several pharmaceuticals in the Watch list of substances for Union-wide monitoring
G2.2	Article 9 of Directive (EU) 2024/3019 introduces an extended producer responsibility (EPR) scheme for producers of medicinal products for human use and cosmetic products, aimed at covering at least 80% of the costs of the quaternary treatment and the monitoring of micropollutants
G2.3	Article 14 of Directive (EU) 2024/3019 requires that discharges of non-domestic wastewater into collecting systems and urban wastewater treatment plants are subject to prior regulations and/or specific authorisations ensuring that: the water quality requirements set out in other Union legislation are fulfilled; where applicable, the quality and quantity of relevant discharges of non-domestic wastewater are monitored; the released polluting substances do not limit any capacity to recover resources, including the reuse of treated water and the recovery of nutrients or other material from urban wastewater or sludge
G2.4	Directive (EU) 2024/1785 added Article 14a to the Industrial Emissions Directive (2010/75/EU, IED), requiring the operator to have an environmental management system that should include: objectives and performance indicators in relation to significant environmental aspects; a chemicals inventory of hazardous substances; measures taken to achieve the environmental objectives and avoid risks for human health and environment; a transformation plan transformation plan was introduced by Directive (EU) 2024/1785; should contain information on how to transform the installation during the 2030–2050 to contribute to a sustainable, clean, circular, resource-efficient and climate-neutral economy by 2050 (art. 27d IED)
G2.5	Pharmaceuticals are proposed to be included in Part A of Annex I to Directive 2008/105/EC as priority substances in surface water^d^
G2.6	Annex I to Directive 2006/118/EC is proposed to be amended to include pharmaceuticals^d^
**Group 3: New requirements for packaging and public procurement, sustainable products, chemicals and materials**	
G3.1	Packaging placed on the EU market should be manufactured so that substances of concern as constituents of the packaging material or of any of the packaging components are minimised, including their presence in emissions and any outcomes of waste management, such as secondary raw materials, ashes or other material for final disposal (art. 5 of Regulation (EU) 2025/40)
G3.2	All packaging placed on the market should be recyclable (art. 6 of Regulation (EU) 2025/40), a requirement that would apply from January 2030 at the earliest exemptions: immediate packaging of medicinal products and outer packaging if such packaging is necessary to comply with specific requirements to preserve the quality of the medicinal product by 2035, the EC shall review the exemptions and assess the appropriateness of their continuity
G3.3	The EC must adopt implementing acts specifying minimum mandatory requirements for public contracts in which the packaging or packaged products represent more than 30% of the estimated contract value (art. 63 of Regulation (EU) 2025/40)
G3.4	The EC must adopt delegated acts to establish ecodesign requirements for products to improve their environmental sustainability (art. 4 of Regulation (EU) 2024/1781) a specific aspect is (among others) the presence of substances of concern does not apply to human or veterinary medicinal products, but applies to medical devices
G3.5	Contracting authorities should award public contracts complying with the minimum ecodesign requirements set out for the products covered by delegated acts (art. 65 of Regulation (EU) 2024/1781)
G3.6	The EC announced in the Chemicals Strategy for Sustainability^e^ that it will develop ‘safe and sustainable by design’ criteria for chemicals and materials. Commission recommendation establishing a European assessment framework for ‘safe and sustainable by design’ chemicals and materials^f^ stated that the application of the framework will make possible the definition of ‘safe and sustainable by design’ criteria which should help set high standards for the safety and sustainability of chemicals and materials. testing period of this non-legally binding framework ended in 2024, the framework will be revised in 2025 the defining ‘safe and sustainable by design’ criteria in parallel with the revision of the framework
**Group 4: Amendments to the CLP Regulation**	
G4.1	Commission delegated Regulation (EU) 2023/707 amended Annex I of the CLP Regulation to include sections for endocrine disruption for human health (Part 3, Section 3.11), endocrine disruption for the environment (Part 4, Section 4.2), for PBT or vPvB properties (Part 4, Section 4.3), and for persistent, mobile and toxic (PMT) or very persistent, very mobile (vPvM) properties (Part 4, Section 4.4)
G4.2	Regulation (EU) 2024/2865 amended Articles 5 and 6 of the CLP Regulation to incorporate evaluation requirements for these new hazard classes
G4.3	Regulation (EU) 2024/2865 amended Article 36 of the CLP Regulation by adding the new hazard classes to its scope
**Group 5: ‘One substance – one assessment’ approach and the concept of ‘essential use’**	
G5.1	Article 23 of the Regulation (EC) No 178/2002 is proposed to be amended to include an obligation to the European Food Safety Authority (EFSA) to cooperate with other EU agencies (European Medicines Agency (EMA), European Chemicals Agency (ECHA), European Environment Agency (EEA)) on the provision of scientific opinions, on the exchange of data and information, and on the development of scientific methodologies for the assessment of chemicals^g^
G5.2	Article 30 of the Regulation (EC) No 178/2002 is proposed to be amended to strengthen the obligation to resolve diverging scientific opinions between the EFSA and other EU agencies^g^
G5.3	Annex I to Regulation (EU) 2017/745 is proposed to be amended to require that the ECHA updates guidelines on the benefit-risk assessment of the presence of phthalates in medical devices, as well as guidelines on other CMR and endocrine-disrupting substances^g^
G5.4	Annex IV of Directive 2011/65/EU lists applications exempted from the restrictions specific to medical devices. Industry can apply time-limited exemptions. It is proposed to amend Article 5 of the Directive so that an application for granting, renewing or revoking an exemption shall be made to the ECHA^h^
G5.5	It is proposed that the ECHA must establish and manage a common data platform on chemicals^i^ the platform should provide access to all chemicals data generated or submitted as part of the implementation of Union acts listed in Annex I to the proposed Regulation and held by the Agencies or the EC, but as regards data related to human and veterinary medicinal products as part of the implementation of the Union acts listed in Annex I, Part 2, only as far as: the data is held by the EMA; it relates to active substances that are subject to regulatory procedures under other Union acts listed in Annex I, Part 1 or have particular PBT properties or have a known high level of residues in the environment; and it falls into at least one of the following categories: non-clinical safety data, environmental risk assessment (human or veterinary) data, maximum residue levels and data used to derive them the EC can adopt delegated acts to include data related to substances contained in medicinal products besides active substances or active substances with other than PBT properties, or to add new categories of data types no later than six years after the entry into force of the proposed Regulation the EC must assess and adopt a report on the appropriateness and cost-benefit balance of including the following data relating to medicinal products: new categories of data types, data on substances other than active substances, data on active substances with properties not yet referred to in the proposed Regulation, data collected and submitted in the context of Union acts listed in Annex 1, Part 2, and held by national authorities
G5.6	It is proposed that researchers or research consortia funded by Union framework programmes should make available to the ECHA any environmental sustainability related data they collect or generate and to the EEA any human biomonitoring data they collect or generate^i^
G5.7	It is proposed that the EEA and the ECHA should establish, operate, maintain and update a framework of indicators to monitor chemical pollution throughout a chemical’s lifecycle, including emissions, occurrence and fate, to monitor the drivers and impacts of exposure to chemicals, and to measure the effectiveness of chemicals legislation and the transition towards the production of safe and sustainable chemicals^i^ in collaboration with e.g. EMA
G5.8	It is proposed that the EEA should also establish, operate and maintain a Union early warning system for emerging chemical risks^i^ the other EU agencies should identify and gather relevant available data on early warning signals from the field falling within their mandate and provide this data to the EEA
G5.9	Communication on the essential use concept^j^ defines that the use of a most harmful substance is essential for society if the following two criteria are met: (1) that use is necessary for health or safety or is critical for the functioning of society, and (2) there are no acceptable alternatives for uses proven essential, conditions should normally be set to minimise emissions and the exposure of humans and the environment substitution plans with commitments, timelines and steps envisaged towards transitioning alternatives could be required for uses of substances that are deemed essential the concept of essential use only has legal effect when introduced into specific legislation
**Group 6: Restrictions on specific substances**	
G6.1	Article 3 of the Commission Regulation (EU) 2022/63 requires the EC to review the necessity to maintain titanium dioxide (E 171) or to delete it from the Union list of food additives for the exclusive use as colour in medicinal products in Part B of Annex II to Regulation (EC) No 1333/2008 within three years after the date of entering into force of the Regulation (EU) 2022/63^k^
G6.2	Cyclosilaxanes D4, D5 and D6 are proposed to be included in Annex B to the Stockholm Convention on Persistent Organic Pollutants, because they can be considered to meet the screening criteria for persistence, bioaccumulation, long-range transport and adverse effects, and their use is likely to lead to significant adverse human health and environmental effects^l^
G6.3	Regulation (EU) 2024/1849 amended Article 10 of Regulation (EU) 2017/852 to prohibit the use of dental amalgam in the EU from 1 January 2025, except when deemed strictly necessary by the dental practitioner based on the specific medical needs of the patient
G6.4	Mercury is also used in vaccines (in the production process or as a preservative). Regulation (EU) 2024/1849 amended Article 19 of Regulation (EU) 2017/852 to require the EC to report, by 31 December 2029, to the European Parliament and to the Council on the need to phase out remaining mercury uses
**Group 7: Sustainability reporting, sustainable corporate governance and financing, and critical raw materials**	
G7.1	Directive (EU) 2022/2464 amended Articles 19a and 29a of Directive 2013/34/EU to require large undertakings to report information necessary to understand: the undertaking’s impact on sustainability matters; how sustainability matters affect the undertaking’s development, performance and position information about value chain should be included undertakings should report in accordance with the sustainability reporting standards
G7.2	Commission delegated Regulation (EU) 2023/2772 contains the first set of common European Sustainability Reporting Standards that undertakings are to use for carrying out their sustainability reporting
G7.3	Commission delegated Regulation (EU) 2023/2486 sets technical screening criteria for different economic activities regarding environmental sustainability aspects, including: manufacture of plastic packaging, including pharmaceutical packaging (art. 2, Annex II) manufacture of active pharmaceutical ingredients (API) or active substances (art. 3, Annex III) manufacture of medicinal products (art. 3, Annex III)
G7.4	Articles 5 and 6 of Directive (EU) 2024/1760 require Member States to ensure that companies conduct risk-based human rights and environmental due diligence by: integrating due diligence into their policies and risk management systems; identifying and assessing actual or potential adverse impacts and where necessary, prioritising potential and actual adverse impacts; preventing and mitigating potential adverse impacts, and bringing actual adverse impacts to an end and minimising their extent; providing remediation for actual adverse impacts; carrying out meaningful engagement with stakeholders; establishing and maintaining a notification mechanism and complaints procedure; monitoring the effectiveness of their due diligence policy and measures; publicly communicating on due diligence
G7.5	Member States must ensure that companies adopt and put into effect a transition plan for climate change mitigation (art. 22 of Directive (EU) 2024/1760)
G7.6	Member States must ensure that compliance with the obligations resulting from the provisions of national law transposing Directive (EU) 2024/1760, or their voluntary implementation, qualifies as an environmental or social aspect that contracting authorities may consider as part of the award criteria for public and concession contracts, and as an environmental or social condition that contracting authorities may lay down in relation to the performance of public and concession contracts (art. 31)
G7.7	Several substances used in the pharmaceutical sector (such as bismuth, platinum group metals, silicon and titanium) are on the list of strategic raw materials in Annex I of the Regulation (EU) 2024/1252

^a^ EC (2022a).

^b^ European Chemicals Agency (ECHA, 2023a). In August 2025, an updated proposal was published, retaining the provisions of G1.4; see ECHA (2025a). However, a new sector ‘other medical applications’ was added, which now covers excipients, immediate packaging and drug delivery devices. Some uses previously assessed as part of ‘medical devices’ sector (such as fluoropolymers in blisters) are now moved to this new sector. On 27 August 2025, the ECHA released a note stating that its scientific committees will not carry out a sector specific evaluation for eight new sectors (including ‘other medical applications’) in the ongoing restriction procedure (ECHA, 2025b).

^c^ This was repealed by Commission implementing decision (EU) 2025/439, which applies from 2 March 2025. The document analysis in this article was carried out before March 2025, using the 2022 decision.

^d^ EC (2022b).

^e^ EC (2020a).

^f^ EC (2022c).

^g^ EC (2023a).

^h^ EC (2023b).

^i^ EC (2023c).

^j^ EC (2024).

^k^ In August 2025, the EC decided that the use of TiO_2_ as a colour in medicinal products should be maintained (EC, 2025).

^l^ ECHA (2023b). The EC withdrew this proposal in July 2025.

## Results

### EU chemicals policy documents

Initial screening of the documents listed in Supplemental Table S1 identified over 30 initiatives. To narrow the scope of the study, initiatives related to climate, pesticides and biocides were excluded. The European Federation of Pharmaceutical Industries and Associations (EFPIA) was consulted at this stage and a few initiatives were added based on their comments. The resulting 25 initiatives were grouped under seven thematic groups: amendments to REACH ((EC) No 1907/2006); EU water and industrial emissions legislation; new requirements for packaging and public procurement, sustainable products, chemicals and materials; amendments to the CLP Regulation ((EC) No 1272/2008); ‘one substance – one assessment’ approach and the concept of ‘essential use’; restrictions on specific substances; sustainability reporting, sustainable corporate governance and financing, and critical raw materials. Supplemental Table S3 lists the initiatives under the thematic groups. These initiatives were referred to in eight chemicals policy documents and/or their annexes, marked with an asterisk in Supplemental Table S1. The initiatives aim to achieve the objectives of the EU chemicals policy towards green transformation, such as CE and a toxic-free environment, as well as more transparent and consistent chemicals safety assessments, and sustainability throughout the value chain. The second step was to identify concrete changes made or proposed to EU chemicals legislation or its implementation from the initiatives. Table 1 lists these changes under the different themes.

### EU pharmaceutical policy and legislation

#### Overview

Five policy documents and nine legislation and guidance documents referred to the changes listed in Table 1 either directly or indirectly. The five policy documents are: EU strategic approach to pharmaceuticals in the environment (EC, 2019c, hereinafter ‘PiE strategy’); update on progress and implementation of the PiE strategy (EC, 2020b); pharmaceutical strategy for Europe (EC, 2020c); reform of the pharmaceutical legislation and measures addressing antimicrobial resistance (EC, 2023d); addressing medicine shortages in the EU (EC, 2023e). The nine legislation and guidance documents are: Regulation (EU) 2017/745; Regulation (EU) 2017/746; Regulation (EU) 2019/6; Regulation (EU) 2021/522; Council recommendation on stepping up EU actions to combat antimicrobial resistance in a One Health approach (Council of the European Union, 2023); guideline on the environmental risk assessment of medicinal products for human use (European Medicines Agency [EMA], 2024a); proposal for a Directive on the Union code relating to medicinal products for human use (EC, 2023f) (hereinafter ‘proposed MPH Directive’); proposal for a Regulation laying down Union procedures for the authorisation and supervision of medicinal products for human use and establishing rules governing the EMA (EC, 2023g); and update of the guidelines on the benefit-risk assessment of the presence of phthalates in certain medical devices (Scientific Committee on Health, Environmental and Emerging Risks [SCHEER], 2024).

All thematic groups were referred to, but not evenly (Table 2). A total of 63 quotations were found in the fourteen documents. Some quotations referred to several changes, so the sum of the numbers in parentheses in Table 2 does not equal the total number of quotations. Groups two and four were referred to most often, while groups six and three were referred to only twice and three times, respectively. All groups, except group six, were also referred to in some of the quotations by broader wording that referred to the group in general, but not explicitly to any change. These quotations are included in Table 2 under the category Gx.gen, where ‘x’ is the group number. The main points of general quotations are described below, along with the quotations related to specific changes in each group.

**Table 2.** The changes coded in Table 1 that were referred to in EU pharmaceutical policy, legislation and guidance documents. Number of quotations in parentheses.

Group	Code									
**1**	**G1.1**	**G1.2**	**G1.3**	**G1.4**	**G1.gen**					
	X (8)	–	–	–	X (2)					
**2**	**G2.1**	**G2.2**	**G2.3**	**G2.4**	**G2.5**	**G2.6**	**G2.gen**			
	X (7)	X (5)	X (1)	X (5)	X (8)	X (6)	X (16)			
**3**	**G3.1**	**G3.2**	**G3.3**	**G3.4**	**G3.5**	**G3.6**	**G3.gen**			
	X (1)	X (1)	–	–	–	–	X (1)			
**4**	**G4.1**	**G4.2**	**G4.3**	**G4.gen**						
	X (18)	–	X (7)	X (2)						
**5**	**G5.1**	**G5.2**	**G5.3**	**G5.4**	**G5.5**	**G5.6**	**G5.7**	**G5.8**	**G5.9**	**G5.gen**
	–	X (1)	–	–	X (2)	–	–	–	–	X (3)
**6**	**G6.1**	**G6.2**	**G6.3**	**G6.4**						
	X (2)	–	–	–						
**7**	**G7.1**	**G7.2**	**G7.3**	**G7.4**	**G7.5**	**G7.6**	**G7.7**	**G7.gen**		
	–	–	–	–	–	–	X (1)	X (7)		

Note: ‘X’ = referred to; ‘–’ = not referred to.

#### Quotations related to specific changes to EU chemicals legislation

***Groups one and four*.** Groups one and four are treated together, as many of the quotations were for both groups. The first change in group one (G1.1) relating to endocrine disrupting phthalates resulted in eight quotations (Table 2), seven of which also referred to group four changes G4.1 and G4.3. All were found in pharmaceutical legislation and guidance documents concerning medical devices (Regulation (EU) 2017/745), in vitro diagnostic medical devices (Regulation (EU) 2017/746) or SCHEER’s related guidelines (SCHEER, 2024). The seven quotations related to G1.1, G4.1 and G4.3 referred to these changes only indirectly. Both Regulation (EU) 2017/745 and Regulation (EU) 2017/746 require that a device must meet the general safety and performance requirements. For example, Regulation (EU) 2017/745 states that:

Devices, or those parts thereof or those materials used therein that:

— are invasive and come into direct contact with the human body,

— (re)administer medicines, body liquids or other substances, including gases, to/from the body, or

— transport or store such medicines, body fluids or substances, including gases, to be (re)administered to the body, shall only contain the following substances in a concentration that is above 0,1% weight by weight (w/w) where justified … :

… 

(b) substances having endocrine-disrupting properties for which there is scientific evidence of probable serious effects to human health and which are identified either in accordance with the procedure set out in Article 59 of Regulation (EC) No 1907/2006 … . (Regulation (EU) 2017/745 Annex I, Chapter II, point 10.4.1)

Regulation (EU) 2017/745 also requires that the relevant scientific committee must update guidelines on a benefit-risk assessment of the presence of phthalates in medical devices (Annex I, Chapter II, point 10.4.3). The SCHEER updated these guidelines in 2024. According to the guidelines:

Phthalates currently classified as reproductive toxicants category 1B under the Classification, Labelling and Packaging (CLP) Regulation … and identified as substances of very high concern (SVHC) under Article 57(c) of Regulation (EC) 1907/2006 (REACH) are listed in Annex 5 of this document. This list may continuously be updated, so it is recommended to consult the Annex VI of the CLP Regulation when using a CMR/ED phthalate as constituent in a medical device. (SCHEER, 2024, p. 12)

The guidelines also referred directly to Commission Regulation (EU) 2021/2045 and G1.1 as follows:

Regarding the exemption of medical devices from the REACH restriction requirements several changes have been published recently. Regulation (EU) 2021/2045 … extended the scope of use of several phthalates in the EU, including that of DEHP. Since this modification of entry n°4 of the REACH authorisation list to include DEHP's endocrine disrupting (ED) properties, use of DEHP in medical devices (previously exempt from the REACH authorisation) will be subject to an authorisation requirement. (SCHEER, 2024, p. 67)

This quotation was only for G1.1, not the changes in group four. The SCHEER noted that Commission Regulation (EU) 2023/2482 extended the deadline for the use of DEHP in medical devices until July 2030 (SCHEER, 2024).

The SCHEER guidelines included also quotations referring only to G4.1. Commission Delegated Regulation (EU) 2023/707 was directly referred to:

Recently, the Commission Delegated Regulation (EU) 2023/707 amended Regulation (EC) 1272/2008 (CLP), introducing two hazard categories for endocrine disruptors for human health with Category 1 considering known or presumed endocrine disruptors for human health, and Category 2 considering suspected endocrine disruptors for human health. (SCHEER, 2024, p. 13)

The guidelines emphasise that endocrine disruption is an essential part of hazard characterisation and can be described according to the guidance published by EFSA and ECHA (SCHEER, 2024). However, the SCHEER noted that:

As the MDR was published in 2017, there is no reference to this ED categorisation. This ED categorisation is also not yet included in the Medical Device Coordination Group (MDCG) endorsed documents or other EU Guidance documents on medical devices. (SCHEER, 2024, p. 25)

The EMA environmental risk assessment (ERA) guideline also implicitly referred to G4.1. It was stated that:

For industrial chemicals, the criteria for the assessment of P, B and T properties … are specified in REACH Annex XIII. However, the specific classifications for the T-criterion … are applicable only for chemicals already classified under REACH according to the Regulation EC No 1272/2008 … . They are not used in the context of preclinical hazard assessment for human pharmaceuticals, as human pharmaceuticals are not within the scope of the CLP Regulation. (EMA, 2024a, p. 53)

The EMA explains that:

Harmonised classifications in the CLP inventory can be used to conclude on the T criterion. In case the substance fulfils the T criterion based on classification in the CLP inventory, no additional toxicity testing is needed for the PBT assessment. (EMA, 2024a, p. 57)

Proposals for MPH Directive and Regulation laying down Union procedures for the authorisation and supervision of medicinal products for human use also refer to G4.1 indirectly. Article 22 of the MPH Directive sets out that:

1. When preparing the environmental risk assessment (‘ERA’) … , the applicant shall take into account the scientific guidelines on the environmental risk assessment of medicinal products for human use … 

2. The ERA shall indicate whether the medicinal product or any of its ingredients or other constituents is one of the following substances according to the criteria of Annex I to the Regulation (EC) No 1272/2008: (a) persistent, bioaccumulative and toxic (PBT); (b) very persistent and very bioaccumulative (vPvB); (c) persistent, mobile and toxic (PMT), very persistent and very mobile (vPvM); or are endocrine active agents. (EC, 2023f, pp. 62–63)

The proposed Regulation refers to the requirements of the MPH Directive, including these ERA requirements (EC, 2023g). Article 51(1)(f) of the MPH Directive also states that a medicinal product should be subject to prescription if it:

[C]ontains an active substance which are [*sic*] persistent, bioaccumulative and toxic, or very persistent and very bioaccumulative, or persistent, mobile and toxic, or very persistent and very mobile for which medical prescription is required as risk minimisation measure with regard to the environment, unless the use of the medicinal product and the patient safety require otherwise. (EC, 2023f, p. 85)

The changes G1.2–G1.4 and G4.2 were not referred to in the documents examined. The two general quotations in group one (G1.gen) noted the links between chemicals and pharmaceutical legislation as regards environmental issues. General quotations in group four (2) were both from the EMA ERA guideline and emphasised that the guidelines provided in the chemicals legislation (such as REACH guidance) should be followed. The general quotations in group four were common with group two.

***Group two.*** This group received the most quotations, and a high number (16) of general quotations (Table 2). Two of the general quotations were common with group four (see above). Four were common with group seven. They highlighted the need for innovation to attain environmentally sustainable pharmaceuticals and manufacturing, the need to reduce emissions along the supply chain, and the need to strengthen the ERA of medicinal products. The rest (10) of the general quotations stressed the limited monitoring of pharmaceuticals in the environment; the need to take the environment into account from a lifecycle perspective; the potential of procurement as an instrument to steer the industry towards greener design and manufacturing; and One Health approach and the need to tackle AMR.

Four quotations in the pharmaceutical policy documents referred to the surface water Watch list (G2.1), stating that several pharmaceuticals have already been included in the previous list (year 2020) and that new pharmaceuticals were considered for the 2022 list. The documents that referred to G2.1 were the PiE strategy and a report on its progress (EC, 2020b) that were published before the 2022 Watch list was available. Pharmaceutical legislation documents included three quotations:

Where there is concern that a pharmaceutical substance could pose serious risk to the environment, it may be appropriate to examine that substance in the context of Union environmental legislation. In particular, under Directive 2000/60/EC … , it may be appropriate to identify whether that substance is a substance for inclusion in the surface water watch list, in order to gather monitoring data on it. It may be appropriate to include it in the list of priority substances and to set an environmental quality standard for it, as well as to identify measures to reduce its emissions to the environment. (Regulation (EU) 2019/6, recital 32)

3. The applicant shall also include in the ERA risk mitigation measures to avoid or where it is not possible, limit emissions to air, water and soil of pollutants listed in Directive 2000/60/EC, Directive 2006/118/EC, Directive 2008/105/EC and Directive 2010/75/EU … . (EC, 2023f, p. 63)

6. The marketing authorisation holder shall update the ERA … , if new information … becomes available and could lead to a change of the conclusions of the ERA. The update shall include any relevant information from environmental monitoring, including monitoring under Directive 2000/60/EC, … . (EC, 2023f, p. 63)

The EPR scheme (G2.2) resulted in five quotations. All were in pharmaceutical policy documents and referred to the revision of the urban wastewater treatment directive. Concerns about the spread of AMR were highlighted:

The fact that antimicrobials have been detected in wastewater treatment, manufacturing effluent, surface and ground waters is of particular concern, as their presence boosts antimicrobial resistance … . Pharmaceuticals present in the environment are affecting not only the environment; if they enter the water cycle or the food chain, they also affect human health directly. Such negative effects have been considered in the recently adopted Commission proposal for the Urban Waste Water Treatment directive, which includes an Extended Producer Responsibility scheme that also applies to pharmaceuticals … . (EC, 2023d, p. 13)

The requirement for a prior authorisation for non-domestic wastewater discharges (G2.3) was only indirectly referred to in one quotation that was common with G2.2:

[A]s as part of the impact assessment for the potential revision of the Urban Waste Water Treatment Directive, ways will be explored to tackle micro-pollutants, including pharmaceuticals, more effectively … . (EC, 2020b, pp. 4–5)

The change G2.4 resulted in five indirect quotations, two of which related to ERA and one to recital 32 of Regulation (EU) 2019/6 were common with G2.1 (see above). Recital 32 of Regulation (EU) 2019/6 specifies, as regards measures to reduce the emissions of pharmaceuticals in the environment, that:

Those measures could include measures to reduce emissions from manufacturing by following Best Available Techniques (BAT) under Directive 2010/75/EU … , particularly if the emission of active pharmaceutical ingredients have been identified as a key environmental issue during the drafting or revision of relevant Best Available Technique Reference Documents (BREFs) and their accompanying BAT conclusions. (Regulation (EU) 2019/6, recital 32)

The two quotations referring solely to G2.4 were in pharmaceutical policy documents, referring to the BREFs and the possibility of considering the release of pharmaceuticals into water bodies when the IED is reviewed.

The changes G2.5 and G2.6 resulted in eight and six quotations, respectively. All the quotations on G2.6 were common to G2.5. They referred to the proposal amending EU water directives (EC, 2022b) and the need to consider pharmaceuticals in the revision of EU water legislation, as the following example shows:

Pharmaceuticals are being considered in the ongoing revision process of the Priority Substances List under the Water Framework Directive and [*sic*] well as the lists of substances annexed to the Groundwater Directive. (EC, 2020b, p. 9)

Reference was also made to the inclusion of pollutants listed in the EU water legislation in the ERA. These quotations relating to ERA requirements were common with G2.1 and G2.4 (see above). The two quotations that referred to G2.5 but not G2.6 were recital 32 of Regulation (EU) 2019/6 (see the quotation related to G2.1 above) and the following quotation:

Substances for which enough information is available could be included in the Priority Substances List under the Water Framework Directive … . (EC, 2020b, p. 17)

***Group three.*** The only quotation referring to specific changes in group three was:

[U]nder the environmental legislation the packaging and packaging waste legislation is under review focusing – among others – on reducing (over)packaging and reinforcement of essential requirements for all packaging in general, accommodating however sector specific requirements, such as the requirements for pharmaceutical packaging. (EC, 2020b, p. 14)

The quotation was in the PiE strategy update document (EC, 2020b), so although it referred to the general sustainability requirements introduced by the changes G3.1 and G3.2, the wording was very general. The other changes (G3.3–G3.6) were not referred to, but pharmaceutical strategy for Europe included the following general quotation that was common to group seven:

Actions in the area of **public procurement** can foster competition and improve access. Public buyers should design smart and innovative procurement procedures, e.g. by assessing the role of ‘winner-takes it all’ procedures and improving related aspects (such as price conditionality, timely delivery, ‘green production’ and security and continuity of supply) … . (Bold in original) (EC, 2020c, p. 7)

***Group five.*** This group also had few quotations. Reference was made to a common data platform (G5.5):

The Chemicals Strategy proposes several actions as regards the ‘one substance, one assessment’ approach as introduced by the European Green Deal, which include the development of a common open data platform to be implemented for chemicals. (EC, 2020b, p. 12)

It was recurred in the footnote related to this quotation that ‘this will be without prejudice to the product specific legislation requirements’ (EC, 2020b, p. 12). The second quotation for G5.5 referred to both G5.2 and G5.5. It was in Article 139 of the proposed Regulation laying down Union procedures for the authorisation and supervision of medicinal products for human use:

1. The Agency shall take the necessary and appropriate measures to monitor and identify at an early stage any potential source of divergence between its scientific opinions and the scientific opinions issued by other Union bodies and agencies carrying out similar tasks in relation to issues of common concern … .

5. … [T]he Agency shall make arrangements with other bodies or agencies established under Union law for cooperation on scientific assessments and methodologies. The Agency shall also make arrangements for the exchange of data and information on relevant substances with the Commission, Member States’ authorities and other Union Agencies, in particular for environmental risk assessments, … . (EC, 2023g, p. 126)

Pursuant to Article 139(5) the EMA should prepare for data and information exchange with other EU agencies – reflecting amendment G5.5. Article 139(1) is linked to G5.2, the obligation to resolve diverging scientific opinions between different Union bodies. The other changes (G5.1, G5.3, G5.4, G5.6–G5.9) were not referred to. General references to this group (3) noted the ‘one substance, one assessment’ approach and its aim to facilitate cooperation between EU agencies.

***Group six.*** There were only two quotations, both for titanium dioxide (though not explicitly named) as a colour in medicinal products (G6.1). The changes G6.2–G6.4 were not referred to. The recitals of the proposed MPH Directive explain the current regulatory status of the use of colours in human and veterinary medicinal products (EC, 2023f), and note the need to address the possible ban on food additives:

[I]t is also appropriate to foresee a specific assessment for the use of the colour in medicines when a food additive is removed from Union list of food additives. Therefore, in this specific case, EMA should carry out its own assessment for the use of the colour in medicines, taking into account the EFSA opinion and its underlying scientific evidence, as well as any additional scientific evidence and giving particular consideration to the use in medicines. (EC, 2023f, p. 37)

This is further clarified in Article 27:

2. Colours shall be used in medicinal products only if they are included in one of the following lists: (a) the Union list of authorised food additives in Table 1 in Part B of Annex II to Regulation (EC) No 1333/2008 … ; (b) the list established by the Commission pursuant to paragraph 3.

3. The Commission may establish a list of colours permitted for use in medicinal products other than those included in the Union list of authorised food additives.

The Commission shall, where applicable on the basis of an opinion of the Agency, adopt a decision whether the colour concerned shall be added to list of colours permitted for use in medicinal products referred to in the first subparagraph.

A colour may be added to the list of colours permitted for use in medicinal products only where the colour has been removed from the Union list of authorised food additives. (EC, 2023f, p. 67)

***Group seven.*** The changes G7.1–G7.6 were not referred to. For change G7.7, one quotation was identified:

As an element in relation to supply chains, the proposed Critical Raw Material Regulation will secure the availability of certain materials relevant for the production of medicines. (EC, 2023d, p. 6)

General quotations that only referred to group seven (2) emphasised security of supply. The first stated that:

Coordination at EU level could offer a strategic frame to enhance security of supply of the identified critical medicines through ***public procurement***. This could draw on Commission guidance and common criteria for the procurement of critical medicines, such as green production … . (Bold and italics in original) (EC, 2023e, p. 12)

The EC will also seek strategic partnerships with third countries for production of critical medicines and APIs, noting that:

These actions could be tailor-made to reflect the potential of different partners to help secure supply or whether a third country requires additional monitoring, prevention and minimisation of environmental, social or legal impacts. (EC, 2023e, p. 16)

#### Topics arising from the quotations

Inductive analysis revealed seven topics from the quotations: substances having endocrine-disrupting properties; prescription; titanium dioxide; ‘one substance, one assessment’ approach; EU water legislation; One Health approach and AMR; and public procurement and security of supply.

***Endocrine-disrupting substances and titanium dioxide.*** Substances having endocrine-disrupting properties were considered mainly in pharmaceutical legislation and guidance documents. Most of the quotations focused on phthalates, particularly their use in medical devices, in vitro diagnostic medical devices, and related SCHEER guidelines. The guidelines stressed that the use of DEHP in medical devices will be banned after July 2030, unless REACH authorisation is applied before January 2029 (SCHEER, 2024). The SCHEER also highlighted that though there are currently no references to new hazard classification in medical devices regulations, endocrine disruption is an essential part of hazard characterisation and attention should be given to endocrine-disrupting properties of the possible alternative substances. The EMA ERA guideline also addressed this topic by stating that the CLP Regulation can be used to assess whether a toxicity criterion is met (EMA, 2024a). This is not restricted to phthalates, but all chemicals that are already classified under the CLP Regulation. The change in chemicals legislation regarding titanium dioxide was reflected in the proposed MPH Directive (EC, 2023f). The possible ban was addressed as explained above (see Group six).

***Prescription.*** The proposed MPH Directive requires the use of prescription as a risk mitigation measure as regards the environment, if a medicinal product contains an API that is PBT, vPvB, PMT, or vPvM (EC, 2023f). However, this requirement only applies ‘unless the use of the medicinal product and the patient safety require otherwise’ (EC, 2023f, p. 85). It should be noted that the proposed MPH Directive requires antimicrobials to be prescription-only, irrespective of their use.

*‘**One substance, one assessment’*** ***approach.*** The proposed Regulation laying down Union procedures for the authorisation and supervision of medicinal products for human use calls for increased cooperation between Union agencies, for example on scientific opinions and assessment methods (EC, 2023g). This requires the exchange of information between agencies, including the development of a common data platform (EC, 2023c). However, in the provisional agreement resulting from interinstitutional negotiations on the proposed Regulation concerning the data platform, data related to human and veterinary medicinal products is considerably limited (see Table 1 and EC, 2023c).

***EU water legislation, One Health approach and AMR.*** Pharmaceutical policy and legislation (see Group two above) acknowledged the need to consider pharmaceuticals in the revision of EU water legislation. One Health approach and the need to tackle AMR were reflected in two documents (Council of the European Union, 2023; EC, 2023d). It was highlighted that if pharmaceuticals enter the water cycle or food chain, they also affect human health directly (EC, 2023d), requiring the One Health approach. The EPR scheme was identified as one possible measure to address the AMR.

***Public procurement and security of supply.*** Public procurement and security of supply were reflected together, for example pharmaceutical strategy for Europe stated that public procurement can enhance ‘“green production” and security and continuity of supply’ (EC, 2020c, p. 7). It was also underlined that strategic partnerships with third countries could be used not only to secure supply but also to address environmental concerns in supply chains (EC, 2023e).

## Discussion

### Cross-cutting structural changes in EU political sphere of chemicals

The first research question examined which recent changes in EU chemicals policy and legislation – supporting green transformation – may affect the pharmaceutical sector, and identified concrete changes to EU chemicals legislation. Eight chemicals policy documents and/or their annexes (Supplemental Table S1) resulted in 25 concrete changes included in this study. The policy documents contained strategies focusing on industry and CE, as well as strategies emphasising the conservation of biodiversity and the reduction of pollution.

The identified concrete changes revealed that structural changes in EU political sphere of chemicals can induce a broad range of implications to pharmaceutical sector (Table 1). For example, these changes address endocrine disruptors, microplastics and PFASs. Though human and veterinary medicinal products are often exempt from these provisions, new reporting requirements have been introduced, and it should be noted that many of these new provisions apply to medical devices. Increased reporting is also required, for example, in relation to the EPR scheme, environmental management system, sustainability reporting, and human rights and environmental due diligence processes. Member States and national authorities will monitor emissions from the pharmaceutical sector more closely in the future, and EU agencies should collaborate on scientific opinions, data and information exchange, and chemical assessment. It is also indicated that public procurement should support the uptake of certain changes, and that the EC and/or Member States should take measures to enable this.

The results showed that the concrete changes addressed the objectives of the EGD and accompanying policies on toxic-free environment, enhancing cooperation between EU agencies, accelerating the CE, and improving the sustainability of value chains. It is noteworthy that changes in EU chemical policy and legislation recognise the need for derogations in certain cases for human and veterinary medicinal products, but, in general, no specific approach is applied that would exempt the health sector entirely from the application of the provisions. This can be seen as a move towards transformation governance. As O’Brien (2018) has pointed out, when structures (norms, rules, regulations, institutions) are changed with the aim of influencing a larger system (for example, the assessment or regulation of chemicals across different sectors in the EU), these systemic changes may facilitate changes in the practical sphere. Scheringer and Schulz (2025) have recently argued that society needs a more comprehensive approach to chemicals (they use the term ‘chemicals transition’) in order to reverse the unfavourable trend in chemical safety, which they say is leading in the same direction as the climate crisis. However, the cross-cutting structural changes in EU political sphere of chemicals may also constrain practical responses to transformation (O’Brien, 2018), so it is important to examine how the changes are reflected in the EU political sphere of health, which we will do next.

### Fragmented reflections within the EU political sphere of health

The second research question analysed how the changes to EU chemicals legislation or its implementation are reflected in EU pharmaceutical policy, legislation and related guidance. Overall, these changes were rarely referred to in pharmaceutical policy, legislation and guidance (Table 2). Many of the changes did not result in any quotations in pharmaceutical policy, legislation, and guidance documents examined. This may partly be because some changes are still at the proposal stage, and many are new legislation that is in the process of being implemented. Pharmaceutical legislation and guidance may later be adapted to these upcoming changes. However, one explanation for the scarcity of reflections is the prevalence of institutional silos between different sectors in EU regulatory system (see, for example EFPIA, 2023a, 2023b; Evans et al., 2016). Evans et al. (2016) identified different regulatory silos for chemicals in the EU and argued that chemical regulation for the protection of human health must transcend the silos between different regulatory areas, such as pharmaceutical legislation, industrial emissions legislation, and water legislation. On the contrary, the pharmaceutical industry underlines that medicinal products (in particular medicines for human use) cannot be regulated like other commodities, which ‘calls for a different policy approach than the one being considered for other goods or chemicals’ (EFPIA, 2023a, p. 5). It is acknowledged, however, that legislation from other sectors (for example, environmental, food, chemical) increasingly impacts the development, manufacture and supply of medicines (EFPIA, 2023b). The EFPIA (2023b) notes that, as many legislative initiatives in other sectors are currently under review, there is uncertainty about these impacts.

An inductive analysis of the quotations indicated that some changes to EU chemicals legislation have got more prominence in pharmaceutical policy, legislation and guidance than others. Substances having endocrine-disrupting properties and possible ban of titanium dioxide were addressed. There appears to be ambiguity on how the CLP Regulation and new hazard classifications, for example for endocrine-disrupting properties, should be considered in the ERA. ECHA stated in the updated REACH guidance that Annex I of CLP Regulation was amended to include a possibility to conclude on toxicity based on classification as endocrine disruptor (ECHA, 2023c). The proposed MPH Directive requires that the ERA must indicate ‘whether the *medicinal product or any of its ingredients or other constituents’* (italics here) is an endocrine active agent according to the criteria set in the CLP Regulation (EC, 2023f, p. 63). However, the EMA guidelines noted that human pharmaceuticals are not within the scope of the CLP Regulation (EMA, 2024a). According to the EFPIA’s, 2022 survey, 7% of all 520 notified APIs were expected to have endocrine-disrupting properties related to human health, and a further 12% were suspected of having such effects (EFPIA, 2022). For environmental effects, endocrine-disrupting properties could not be assessed in more than 50% of the cases due to lack of sufficient data.

The proposed MPH Directive includes provisions that may be intended to circumvent a possible ban on colour in medicines when a food additive is removed from Union list of food additives. The titanium dioxide case has probably influenced these provisions. Commission Regulation (EU) 2022/63 required the EC to review the need to maintain the exemption for titanium dioxide as a colour in medicinal products and in August 2025 the EC decided that the use of TiO_2_ as a colour in medicinal products should be maintained (EC, 2025). The EC based its decision on the EMA’s updated analysis on the feasibility of replacing TiO_2_ with alternatives (EMA, 2024b) and emphasised that: TiO_2_ is important for the safety, quality and efficacy of medicinal products; there is a lack of feasible alternatives; there is a risk of shortages, which could be detrimental to patients (EC, 2025). However, the EC reminded that the pharmaceutical industry should continue to adapt to scientific progress and development of new excipients, particularly for new products (EC, 2025). EMA has previously stated that ‘applicants are reminded to make all possible efforts to accelerate the research and development of alternatives and to replace TiO2’ (EMA, 2022). A recent study by Cairat et al. (2024) assessed that in France 38% of pharmaceuticals on the market contained TiO_2_. For capsules and film-coated tablets the percentages were 95% and 92%, respectively.

The analysis also revealed that there is uncertainty on how product-specific requirements can be considered in the ‘one substance, one assessment’ approach. EFPIA has stated that pharmaceutical industry does not oppose the approach, but ‘the impact on medicines has not been fully considered’ and that it ‘is reasonably expected to result in the removal and replacement of chemicals also used in healthcare products’ (EFPIA, 2023b, p. 57). However, although data to be contained in the common data platform is limited at this stage with regard to medicinal products, the EMA and the pharmaceutical sector in general should prepare for broader and more transparent information sharing, taking into account also changes G5.6–G5.9, even though they were not reflected in the documents included in this study. Tampach et al. (2025) have analysed scientific cooperation between EU agencies dealing with One health issues and found that many inter-agency cooperation tools are in place and that the agencies have increasingly shared knowledge and experience.

The identified reflections related to food additives (TiO_2_) and the ‘one substance, one assessment’ approach are examples of possible conflicts between the political and practical spheres across the different sectors. Structural changes in EU political sphere of chemicals clearly have implications for the political sphere of health, as the reflections above show, but there may be conflicts between the desired outcomes in the practical sphere in different sectors, which should be considered in transformation governance. The fundamental objective of the health sector is to ensure human health and access to medicines (EFPIA, 2023a), which understandably leads to actions, strategies and behaviours that promote this outcome in the EU practical sphere of health. If the cross-cutting structural changes in EU political sphere of chemicals threaten to undermine the desired outcomes in the health sector, the responses in the EU political sphere of health to these changes may not necessarily facilitate the green transformation but may even hinder it. O’Brien (2018) stresses the importance of considering the social complexity of transformation processes and the dynamics between practical, personal and political spheres.

Prescription, public procurement and strategic partnerships with third countries were identified as measures that can reduce the environmental impact of pharmaceuticals. Currently, for example, the Swedish Drugs and Therapeutic Committees have included environmental considerations in their prescribing guidelines (Giunchi et al., 2025). Bhullar (2024) has recently examined the compatibility of production-related environmental criteria in public procurement with international trade law and found that they may be compatible. Public procurement provides an extraterritorial regulatory instrument to combat AMR, and Bhullar argues that the principles of EU environmental law – such as the principle of pollution prevention at source and the polluter pays principle – have driven the uptake of green public procurement in the pharmaceutical sector.

Many quotations suggested EU water legislation as a solution to the problem of pharmaceuticals in the environment. However, there are limitations to relying on EU water legislation. The collection of monitoring data is slow, as is the setting of environmental quality standards (Kittery & Miettinen, 2023). Moreover, EU water legislation does not extend beyond EU borders and this approach also increases the complexity of EU legislation, especially in cases of non-compliance. Kittery and Miettinen argued that cross-referencing other EU environmental legislation shifts environmental accountability outside EU pharmaceutical legislation and actors. One Health approach and the need to combat AMR were also highlighted in Group two quotations, and the EPR scheme was mentioned as a specific tool in this context. However, as AMR is a global health threat (Miettinen & Khan, 2022), the impact of the EU EPR system on it globally is likely to be very limited.

### Implications to policy and transformation governance

Scoones et al. (2015) have pointed out – as regards the call for a green transformation – that discussion on transformation is always political, including, for example questions relating to what is to be transformed, who will implement it, or what counts as green. They add that transformations involve various governance challenges, such as which institutions define strategies and conditions for change, or whose rules prevail. O’Brien (2018) has introduced three different spheres (the practical, political, personal) of transformation, the dynamic of which should be taken into account in transformation governance. However, empirical studies on transformation in different spheres are scarce (Amundsen & Hermansen, 2021). This study has contributed to filling this gap by examining the implications of a selected set of EU chemicals policy initiatives, particularly those related to the EGD, as well as concrete changes to EU chemicals legislation on EU pharmaceutical policy and legislation. Empirical evidence on the impact of structural changes introduced in the EU political sphere of chemicals on the EU political sphere of health reveals potential unforeseen responses and may help steer a more coherent transformation governance across different policy sectors in the EU.

### Limitations

This study examined only a limited set of policy and legal documents and related guidance, and did not systematically include other aspects, such as the views expressed in documents produced by various stakeholders. In addition, the aim was not to conduct a comprehensive systematic search of all changes to EU chemicals policy and legislation that may affect the pharmaceutical sector. These are limitations that may restrict the generalisability of the results of this study, but they also indicate future research needs. Future studies should also examine the perceptions of different stakeholders in the pharmaceutical sector (personal sphere, see O’Brien, 2018) on the implications of the structural changes in EU political sphere of chemicals and the need for green transformation in the pharmaceutical sector. This would provide a more holistic understanding of the depth of the needed transformation.

## Conclusions

The first research question of this study examined which recent changes in EU chemicals policy and legislation that support green transformation may affect the pharmaceutical sector, and identified concrete changes to EU chemicals legislation. The identified changes imply that structural changes in EU political sphere of chemicals may have a wide range of implications for the pharmaceutical sector. The amendments recognise the need to derogate in certain cases with regard to medicinal products, but they indicate that, in general, the pharmaceutical sector will not be exempted from the application of the new provisions.

The second research question analysed how these changes are reflected in EU pharmaceutical policy, legislation and related guidance. Many changes to EU chemicals legislation were not reflected in the pharmaceutical policy, legislation and guidance documents examined. This is partly because many of the changes are new and still being implemented. In turn, some parts of the pharmaceutical legislation (such as medical devices regulations) were adopted before the changes to the EU chemicals legislation were notified and could not be considered. The reflections revealed, however, that structural changes in EU political sphere of chemicals clearly have implications for the EU political sphere of health. It was also found that the pharmaceutical sector is taking a reactive and defensive approach to changes in EU chemicals legislation, seeking to maintain a siloed approach. This may be partly due to many changes occurring almost simultaneously, creating uncertainty about their combined and cumulative impacts. Moreover, the pharmaceutical sector and the actors involved may not be well aware of all the different aspects of chemicals legislation that may affect them. An inductive analysis of the quotations indicated that some changes to EU chemicals legislation have got more prominence in the EU political sphere of health than others. Conversely, actors in the chemicals sector may not be able to consider all the cross-cutting implications of the proposed changes.

This study aimed to find out whether structural changes in EU political sphere of chemicals influence the political sphere of health. The findings above suggest that a more integrated approach to chemicals in EU legislation is needed to address the observed challenges. This would require close cooperation between the Environment and Health Directorates-General in order to steer a more coherent transformation governance across different policy sectors in the EU. Furthermore, a paradigm shift in the pharmaceutical sector is needed to acknowledge the relevance of environmental considerations and to truly embed them into pharmaceutical legislation and guidelines. Current measures proposed to tackle the problem of pharmaceuticals in the environment, for example in the quotations of this study, remain fragmented and do not ensure environmental accountability.

## Supplementary Material

Supplemental Material S2 - Tables

Supplemental Material S1 - Legislation List
