# Supplementary material for: Crossing silos: how changes in EU chemicals policy and legislation are reflected in its pharmaceutical policy and legislation
Source: J Pharm Policy Pract. 2025 Nov 24;18(1):2587439. doi: 10.1080/20523211.2025.2587439 (PMC12646081; doi:10.1080/20523211.2025.2587439)
Supplement: Supplemental Material S2 - Tables [file JPPP_A_2587439_SM9767.docx]

**Additional file 2**

**Supplementary Tables S1–S3**

**Table S1.** EU chemicals policy documents that were examined. The documents that contained initiatives examined further in this research are marked with an asterisk.

| **Document name** | **Identifier** |
| --- | --- |
| Towards a Comprehensive European Union Framework on Endocrine Disruptors | COM(2018) 734 final |
| *The European Green Deal | COM(2019) 640 final |
| *The European Green Deal Annex | COM(2019) 640 final Annex |
| *A new Circular Economy Action Plan | COM(2020) 98 final |
| *A new Circular Economy Action Plan Annex | COM(2020) 98 final Annex |
| *A New Industrial Strategy for Europe | COM(2020) 102 final |
| *EU Biodiversity Strategy for 2030 | COM(2020) 380 final |
| *EU Biodiversity Strategy for 2030 Annex | COM(2020) 380 final Annex |
| A Farm to Fork Strategy | COM(2020) 381 final |
| A Farm to Fork Strategy Annex | COM(2020) 381 final Annex |
| *Chemicals Strategy for Sustainability | COM(2020) 667 final |
| *Chemicals Strategy for Sustainability Annex | COM(2020) 667 final Annex |
| An Action Plan for the Development of Organic Production | COM(2021) 141 final |
| An Action Plan for the Development of Organic Production Annex | COM(2021) 141 final Annex |
| A New Approach for a Sustainable Blue Economy in the EU | COM(2021) 240 final |
| *EU Action Plan: ‘Towards Zero Pollution for Air, Water and Soil’ | COM(2021) 400 final |
| *EU Action Plan: ‘Towards Zero Pollution for Air, Water and Soil’ Annexes | COM(2021) 400 final Annexes 1 to 2 |
| *EU Soil Strategy for 2030 | COM(2021) 699 final |
| *On Making Sustainable Products the Norm | COM(2022) 140 final |
| Revision of the EU Pollinators Initiative | COM(2023) 35 final |

**Table S2.** EU pharmaceutical policy documents, pharmaceutical legislation and guidelines that were examined.

| **Policy documents** | |
| --- | --- |
| ***Document name*** | ***Identifier*** |
| European Union Strategic Approach to Pharmaceuticals in the Environment | COM(2019) 128 final |
| Update on Progress and Implementation European Union Strategic Approach to Pharmaceuticals in the Environment | European Union (2020)  doi:10. 2779/037747 |
| Pharmaceutical Strategy for Europe | COM(2020) 761 final |
| EU Global Health Strategy | European Union (2022)  doi:10.2875/22652 |
| Building a European Health Union: Reinforcing the EU’s Resilience for Cross-Border Health Threats | COM(2020) 724 final |
| Reform of the Pharmaceutical Legislation and Measures Addressing Antimicrobial Resistance | COM(2023) 190 final |
| Addressing Medicine Shortages in the EU | COM(2023) 672 final |
| The European Health Union: Acting Together for People's Health | COM(2024) 206 final |
| **Legislation and guidance** | |
| ***Document name*** | ***Identifier*** |
| Regulation (EU) 2017/745 of the European Parliament and of the Council On Medical devices | O.J. (L 117) 1 (May 5, 2017) |
| Regulation (EU) 2017/746 of the European Parliament and of the Council On In Vitro Diagnostic Medical Devices | O.J. (L 117) 176 (May 5, 2017) |
| Regulation (EU) 2019/4 of the European Parliament and of the Council On the Manufacture, Placing on the Market and Use of Medicated Feed | O.J. (L 4) 1 (Jan. 7, 2019) |
| Regulation (EU) 2019/6 of the European Parliament and of the Council On Veterinary Medicinal Products | O.J. (L 4) 43 (Jan. 7, 2019) |
| Regulation (EU) 2021/522 of the European Parliament and of the Council Establishing a Programme for the Union’s Action in the Field of Health (‘EU4Health Programme’) for the Period 2021-2027 | O.J. (L 107) 1 (Mar. 26, 2021) |
| Council Recommendation On Stepping up EU Actions to Combat Antimicrobial Resistance in a One Health Approach | O.J. (C 220) 1 (June 22, 2023) |
| Guideline on the Environmental Risk Assessment of Medicinal Products for Human Use | European Medicines Agency EMEA/CHMP/SWP/4447/00 Rev. 1- Corr. (Aug. 22, 2024) |
| Proposal for a Directive of the European Parliament and of the Council on the Union Code Relating to Medicinal Products for Human Use | COM(2023) 192 final |
| Proposal for a Directive of the European Parliament and of the Council on the Union Code Relating to Medicinal Products for Human Use Annexes | COM(2023) 192 final  Annexes 1 to 8 |
| Proposal for a Regulation of the European Parliament and of the Council Laying Down Union Procedures for the Authorisation and Supervision of Medicinal Products for Human Use and Establishing Rules Governing the European Medicines Agency | COM(2023) 193 final |
| Proposal for a Regulation of the European Parliament and of the Council Laying Down Union Procedures for the Authorisation and Supervision of Medicinal Products for Human Use and Establishing Rules Governing the European Medicines Agency Annexes | COM(2023) 193 final  Annexes 1 to 5 |
| Update of the Guidelines on the Benefit-Risk Assessment of the Presence of Phthalates in Certain Medical Devices Covering Phthalates which are Carcinogenic, Mutagenic, Toxic to Reproduction (CMR) or have Endocrine-disrupting (ED) Properties | Scientific Committee on Health, Environmental and Emerging Risks (SCHEER), final version (June 14, 2024) |

**Table S3.** Grouping of the identified initiatives.

| **Group 1: Amendments to REACH^*^** | |
| --- | --- |
|  | Commission Regulation (EU) 2021/2045 |
|  | Commission Regulation (EU) 2023/2055 |
|  | Restrictions Roadmap Under the Chemicals Strategy for Sustainability^a^ |
|  | Annex XV Restriction Report. Proposal for a Restriction of Per- and Polyfluoroalkyl Substances (PFASs)^b^ |
| **Group 2: EU water and industrial emissions legislation** | |
|  | Commission Implementing Decision (EU) 2022/1307 |
|  | Directive (EU) 2024/1785 |
|  | Proposal for a Directive Amending Directive 2000/60/EC Establishing a Framework for Community Action in the Field of Water Policy, Directive 2006/118/EC on the Protection of Groundwater Against Pollution and Deterioration and Directive 2008/105/EC on Environmental Quality Standards in the Field of Water Policy^c^ |
|  | Directive (EU) 2024/3019 |
| **Group 3: New requirements for packaging and public procurement, sustainable products, chemicals and materials** | |
|  | Regulation (EU) 2025/40 |
|  | Regulation (EU) 2024/1781 |
|  | Commission Recommendation Establishing a European Assessment Framework for ‘Safe and Sustainable by Design’ Chemicals and Materials^d^ |
| **Group 4: Amendments to the CLP Regulation** | |
|  | Commission Delegated Regulation (EU) 2023/707 |
|  | Regulation (EU) 2024/2865 |
| **Group 5: ‘One substance – one assessment’ approach and the concept of ‘essential use’** | |
|  | Proposal for a Regulation Amending Regulations (EC) No 178/2002, (EC) No 401/2009, (EU) 2017/745 and (EU) 2019/1021 as Regards the Re-Attribution of Scientific and Technical Tasks and Improving Cooperation Among Union Agencies in the Area of Chemicals. Provisional agreement resulting from interinstitutional negotiations.^e^ |
|  | Proposal for a Directive Amending Directive 2011/65/EU as Regards the Re-Attribution of Scientific and Technical Tasks to the European Chemicals Agency. Provisional agreement resulting from interinstitutional negotiations.^f^ |
|  | Proposal for a Regulation Establishing a Common Data Platform on Chemicals, Laying Down Rules to Ensure that the Data Contained in it are Findable, Accessible, Interoperable and Reusable and Establishing a Monitoring and Outlook Framework for Chemicals. Provisional agreement resulting from interinstitutional negotiations.^g^ |
|  | Communication from the Commission Guiding Criteria and Principles for the Essential Use Concept in EU Legislation Dealing with Chemicals^h^ |
| **Group 6: Restrictions on Specific Substances** | |
|  | Commission Regulation (EU) 2022/63 |
|  | European Union Proposal to List Octamethylcyclotetrasiloxane (D4), Decamethylcyclopentasiloxane (D5) and Dodecamethylcyclohexasiloxane (D6) in Annex B to the Stockholm Convention on Persistent Organic Pollutants^i^ |
|  | Regulation (EU) 2024/1849 |
| **Group 7: Sustainability Reporting, Sustainable Corporate Governance and Financing, and Critical Raw Materials** | |
|  | Directive (EU) 2022/2464 |
|  | Commission Delegated Regulation (EU) 2023/2772 |
|  | Commission Delegated Regulation (EU) 2023/2486 |
|  | Directive (EU) 2024/1760 |
|  | Regulation (EU) 2024/1252 |

Notes: ^*^For details of the legislation referred to, see Additional file 1.

^a^European Commission. (2022). Restrictions Roadmap under the Chemicals Strategy for Sustainability*.* SWD(2022) 128 final. ^b^European Chemicals Agency. (2023). Annex XV restriction report: proposal for a restriction. <https://echa.europa.eu/documents/10162/1c480180-ece9-1bdd-1eb8-0f3f8e7c0c49>.

^c^European Commission. (2022). Proposal for a Directive of the European Parliament and of the Council Amending Directive 2000/60/EC Establishing a Framework for Community Action in the Field of Water Policy, Directive 2006/118/EC on the Protection of Groundwater Against Pollution and Deterioration and Directive 2008/105/EC on Environmental Quality Standards in the Field of Water Policy. COM(2022) 540 final.

^d^European Commission. (2022). Commission Recommendation Establishing a European Assessment Framework for ‘Safe and Sustainable by Design’ Chemicals and Materials, C(2022) 8854 final.

^e^European Commission. (2023). Proposal for a Regulation of the European Parliament and of the Council Amending Regulations (EC) No 178/2002, (EC) No 401/2009, (EU) 2017/745 and (EU) 2019/1021 of the European Parliament and of the Council As Regards the Re-Attribution of Scientific and Technical Tasks and Improving Cooperation Among Union Agencies in the Area of Chemicals. COM(2023) 783 final. Provisional agreement resulting from interinstitutional negotiations, 25 June 2025. 2023/0455 (COD).

^f^European Commission. (2023). Proposal for a Directive of the European Parliament and of the Council Amending Directive 2011/65/EU of the European Parliament and of the Council As Regards the Re-Attribution of Scientific and Technical Tasks to the European Chemicals Agency. COM(2023) 781 final. Provisional agreement resulting from interinstitutional negotiations, 25 June 2025. 2023/0454 (COD).

^g^European Commission. (2023). Proposal for a Regulation of the European Parliament and of the Council Establishing a Common Data Platform on Chemicals, Laying Down Rules to Ensure That the Data Contained in it are Findable, Accessible, Interoperable and Reusable and Establishing a Monitoring and Outlook Framework for Chemicals, COM(2023) 779 final. Provisional agreement resulting from interinstitutional negotiations, 25 June 2025. 2023/0453 (COD).

^h^European Commission. (2024). Communication from the Commission Guiding Criteria and Principles for the Essential Use Concept in EU Legislation Dealing with Chemicals. C(2024) 1995 final.

^i^European Chemicals Agency. (2023). European Union proposal to list Octamethylcyclotetrasiloxane (D4), Decamethylcyclopentasiloxane (D5) and Dodecamethylcyclohexasiloxane (D6) in Annex B to the Stockholm Convention on Persistent Organic Pollutants. <https://echa.europa.eu/documents/10162/96f7a576-20b1-a79b-2eac-3b772ae8e1d4>. The European Commission withdrew this proposal in July 2025.
