# Supplementary material for: Crossing silos: how changes in EU chemicals policy and legislation are reflected in its pharmaceutical policy and legislation
Source: J Pharm Policy Pract. 2025 Nov 24;18(1):2587439. doi: 10.1080/20523211.2025.2587439 (PMC12646081; doi:10.1080/20523211.2025.2587439)
Supplement: Supplemental Material S1 - Legislation List [file JPPP_A_2587439_SM9766.docx]

**Additional file 1**

**List of legislation referred to in this article**

Commission Delegated Regulation (EU) 2023/707 Amending Regulation (EC) No 1272/2008 as Regards Hazard Classes and Criteria for the Classification, Labelling and Packaging of Substances and Mixtures. http://data.europa.eu/eli/reg_del/2023/707/oj

Commission Delegated Regulation (EU) 2023/2486 Supplementing Regulation (EU) 2020/852 of the European Parliament and of the Council by Establishing the Technical Screening Criteria for Determining the Conditions Under Which an Economic Activity Qualifies as Contributing Substantially to the Sustainable Use and Protection of Water and Marine Resources, to the Transition to a Circular Economy, to Pollution Prevention and Control, or to the Protection and Restoration of Biodiversity and Ecosystems and for Determining Whether That Economic Activity Causes No Significant Harm to Any of the Other Environmental Objectives and Amending Commission Delegated Regulation (EU) 2021/2178 as Regards Specific Public Disclosures for Those Economic Activities. http://data.europa.eu/eli/reg_del/2023/2486/oj

Commission Delegated Regulation (EU) 2023/2772 Supplementing Directive 2013/34/EU of the European Parliament and of the Council as Regards Sustainability Reporting Standards. http://data.europa.eu/eli/reg_del/2023/2772/oj

Commission Implementing Decision (EU) 2022/1307 Establishing a Watch List of Substances for Union-wide Monitoring in the Field of Water Policy. http://data.europa.eu/eli/dec_impl/2022/1307/oj

Commission Implementing Decision (EU) 2025/439 Establishing a Watch List of Substances for Union-wide Monitoring in the Field of Water Policy. http://data.europa.eu/eli/dec_impl/2025/439/oj

Commission Regulation (EU) 2021/2045 Amending Annex XIV to Regulation (EC) No 1907/2006 of the European Parliament and of the Council Concerning the Registration, Evaluation, Authorisation and Restriction of Chemicals (REACH). http://data.europa.eu/eli/reg/2021/2045/oj

Commission Regulation (EU) 2022/63 Amending Annexes II and III to Regulation (EC) No 1333/2008 of the European Parliament and of the Council as Regards the Food Additive Titanium Dioxide (E 171). http://data.europa.eu/eli/reg/2022/63/oj

Commission Regulation (EU) 2023/2055 Amending Annex XVII to Regulation (EC) No 1907/2006 of the European Parliament and of the Council Concerning the Registration, Evaluation, Authorisation and Restriction of Chemicals (REACH) as Regards Synthetic Polymer Microparticles. http://data.europa.eu/eli/reg/2023/2055/oj

Commission Regulation (EU) 2023/2482 amending Regulation (EC) No 1907/2006 as regards the substance bis(2-ethylhexyl) phthalate (DEHP) in medical devices. http://data.europa.eu/eli/reg/2023/2482/oj

Directive 2000/60/EC of the European Parliament and of the Council establishing a framework for Community action in the field of water policy. http://data.europa.eu/eli/dir/2000/60/oj

Directive 2006/118/EC of the European Parliament and of the Council on the protection of groundwater against pollution and deterioration. http://data.europa.eu/eli/dir/2006/118/oj

Directive 2008/105/EC of the European Parliament and of the Council on environmental quality standards in the field of water policy. http://data.europa.eu/eli/dir/2008/105/oj

Directive 2010/75/EU of the European Parliament and of the Council on industrial emissions (integrated pollution prevention and control) (recast). http://data.europa.eu/eli/dir/2010/75/oj

Directive 2011/65/EU of the European Parliament and of the Council on the restriction of the use of certain hazardous substances in electrical and electronic equipment (recast). http://data.europa.eu/eli/dir/2011/65/oj

Directive 2013/34/EU of the European Parliament and of the Council on the annual financial statements, consolidated financial statements and related reports of certain types of undertakings. http://data.europa.eu/eli/dir/2013/34/oj

Directive (EU) 2022/2464 of the European Parliament and of the Council Amending Regulation (EU) No 537/2014, Directive 2004/109/EC, Directive 2006/43/EC and Directive 2013/34/EU, as Regards Corporate Sustainability Reporting. http://data.europa.eu/eli/dir/2022/2464/oj

Directive (EU) 2024/1760 of the European Parliament and of the Council On Corporate Sustainability Due Diligence. http://data.europa.eu/eli/dir/2024/1760/oj

Directive (EU) 2024/1785 of the European Parliament and of the Council Amending Directive 2010/75/EU of the European Parliament and of the Council on Industrial Emissions. http://data.europa.eu/eli/dir/2024/1785/oj

Directive (EU) 2024/3019 of the European Parliament and of the Council Concerning Urban Wastewater Treatment (recast). http://data.europa.eu/eli/dir/2024/3019/oj

Regulation (EC) No 178/2002 of the European Parliament and of the Council laying down the general principles and requirements of food law, establishing the European Food Safety Authority and laying down procedures in matters of food safety. http://data.europa.eu/eli/reg/2002/178/oj

Regulation (EC) No 1907/2006 of the European Parliament and of the Council Concerning the Registration, Evaluation, Authorisation and Restriction of Chemicals (REACH). http://data.europa.eu/eli/reg/2006/1907/oj

Regulation (EC) No 1272/2008 of the European Parliament and of the Council On Classification, Labelling and Packaging of Substances and Mixtures. http://data.europa.eu/eli/reg/2008/1272/oj

Regulation (EC) No 1333/2008 of the European Parliament and of the Council on food additives. http://data.europa.eu/eli/reg/2008/1333/oj

Regulation (EU) 2017/745 of the European Parliament and of the Council on medical devices. http://data.europa.eu/eli/reg/2017/745/oj

Regulation (EU) 2017/746 of the European Parliament and of the Council On In Vitro Diagnostic Medical Devices. http://data.europa.eu/eli/reg/2017/746/oj

Regulation (EU) 2017/852 of the European Parliament and of the Council on mercury. http://data.europa.eu/eli/reg/2017/852/oj

Regulation (EU) 2019/6 of the European Parliament and of the Council On Veterinary Medicinal Products and Repealing Directive 2001/82/EC. http://data.europa.eu/eli/reg/2019/6/oj

Regulation (EU) 2021/522 of the European Parliament and of the Council Establishing a Programme for the Union's Action in the Field of Health (‘EU4Health Programme’) for the Period 2021–2027. http://data.europa.eu/eli/reg/2021/522/oj

Regulation (EU) 2024/1252 of the European Parliament and of the Council Establishing a Framework for Ensuring a Secure and Sustainable Supply of Critical Raw Materials. http://data.europa.eu/eli/reg/2024/1252/oj

Regulation (EU) 2024/1781 of the European Parliament and of the Council Establishing a Framework for the Setting of Ecodesign Requirements for Sustainable Products. http://data.europa.eu/eli/reg/2024/1781/oj

Regulation (EU) 2024/1849 of the European Parliament and of the Council Amending Regulation (EU) 2017/852 on Mercury as Regards Dental Amalgam and Other Mercury-Added Products Subject to Export, Import and Manufacturing Restrictions. http://data.europa.eu/eli/reg/2024/1849/oj

Regulation (EU) 2024/2865 of the European Parliament and of the Council amending Regulation (EC) No 1272/2008 on Classification, Labelling and Packaging of Substances and Mixtures. http://data.europa.eu/eli/reg/2024/2865/oj

Regulation (EU) 2025/40 of the European Parliament and of the Council On Packaging and Packaging Waste. http://data.europa.eu/eli/reg/2025/40/oj
